# Spontaneous Ca^2+^ Fluctuations Arise in Thin Astrocytic Processes With Real 3D Geometry

**DOI:** 10.3389/fncel.2021.617989

**Published:** 2021-03-01

**Authors:** László Héja, Zsolt Szabó, Márton Péter, Julianna Kardos

**Affiliations:** ^1^Functional Pharmacology Research Group, Institute of Organic Chemistry, Research Centre for Natural Sciences, Hungarian Academy of Sciences (MTA), Budapest, Hungary; ^2^Hevesy György PhD School of Chemistry, ELTE Eötvös Loránd University, Budapest, Hungary

**Keywords:** astrocyte, Ca^2+^ oscillation, NCX (sodium–calcium exchanger), astrocyte morphology, real geometry, simulation

## Abstract

Fluctuations of cytosolic Ca^2+^ concentration in astrocytes are regarded as a critical non-neuronal signal to regulate neuronal functions. Although such fluctuations can be evoked by neuronal activity, rhythmic astrocytic Ca^2+^ oscillations may also spontaneously arise. Experimental studies hint that these spontaneous astrocytic Ca^2+^ oscillations may lie behind different kinds of emerging neuronal synchronized activities, like epileptogenic bursts or slow-wave rhythms. Despite the potential importance of spontaneous Ca^2+^ oscillations in astrocytes, the mechanism by which they develop is poorly understood. Using simple 3D synapse models and kinetic data of astrocytic Glu transporters (EAATs) and the Na^+^/Ca^2+^ exchanger (NCX), we have previously shown that NCX activity alone can generate markedly stable, spontaneous Ca^2+^ oscillation in the astrocytic leaflet microdomain. Here, we extend that model by incorporating experimentally determined real 3D geometries of 208 excitatory synapses reconstructed from publicly available ultra-resolution electron microscopy datasets. Our simulations predict that the surface/volume ratio (SVR) of peri-synaptic astrocytic processes prominently dictates whether NCX-mediated spontaneous Ca^2+^ oscillations emerge. We also show that increased levels of intracellular astrocytic Na^+^ concentration facilitate the appearance of Ca^2+^ fluctuations. These results further support the principal role of the dynamical reshaping of astrocyte processes in the generation of intrinsic Ca^2+^ oscillations and their spreading over larger astrocytic compartments.

## Introduction

Over the past three decades, astrocytes have emerged as crucial regulators of synaptic function (Zhang et al., 2016). On the cellular scale, many of these regulatory functions operate by controlling the extracellular concentration of various substances pivotal to synaptic activity (Somogyi et al., 1990; Harris et al., 1992; Rusakov et al., 1997, 1998, 1999; Rusakov and Kullmann, 1998a, b; Araque et al., 1999; Bergles et al., 1999; Ventura and Harris, 1999; Newman, 2004; Matsui et al., 2005; Savtchenko and Rusakov, 2007; Heller et al., 2020). One of such classical astrocyte-mediated regulatory function is the uptake of synaptically released glutamate. Glial glutamate uptake by the Na^+^/Glu symporter, Glu transporters (EAATs), in turn, alters astrocytic intracellular Na^+^ concentration, leading to the activation of diverse Na^+^-symporters, like GABA and Gln transporters or Na^+^/K^+^-ATPase (NKA) and Na^+^/K^+^/2Cl^−^ (NKCC1; Lenart et al., 2004; Héja et al., 2009, 2012, 2019; Pál et al., 2013, 2015; Kirischuk et al., 2016; Gerkau et al., 2019; Henneberger et al., 2020; Lerchundi et al., 2020).

Another consequence of the altered astrocytic Na^+^ concentration is the triggering of coupled Ca^2+^ fluctuations (Mergenthaler et al., 2019) mediated mainly by the Na^+^/Ca^2+^ exchanger (NCX; Brazhe et al., 2018). Since NCX operates close to its equilibrium, it can be easily switched between forward and reverse operations (Kirischuk et al., 2016). Moreover, intracellular fluctuations of Na^+^ concentration in the synapse-covering astrocytic microdomain can be intensified by local Na^+^ inhomogeneity due to surface retention of cations by the dipole heads of negatively charged membrane lipids (Breslin et al., 2018). Therefore, EAAT-mediated Glu/Na^+^ symport may easily give rise to local Ca^2+^ fluctuations.

We and others conjectured different Ca^2+^ signaling mechanisms at perisynaptic astrocytic processes (PAPs) and their relevance for the regulation of the tripartite synapses (Kékesi et al., 2015; Kovács et al., 2015; Savtchenko et al., 2015; Kirischuk et al., 2016; Szabó et al., 2017; De Pittà, 2020; Héja and Kardos, 2020; Semyanov et al., 2020). Using a simplified tripartite synapse model built up by geometric modules we have previously shown that NCX alone can generate spontaneous calcium fluctuations, enhanced by glutamate taken up through EAATs (Héja and Kardos, 2020). However, local Na^+^ and Ca^2+^ dynamics in these very thin processes heavily depend on the actual geometry of PAPs. Moreover, this geometry is known to be dynamically changing due to astrocyte activation (Henneberger et al., 2020). Therefore, in the current work, we explored whether NCX activity may introduce rhythmic Ca^2+^ dynamics in real excitatory tripartite synapses using a public annotated database of 1,700 real synapses reconstructed from serial electron microscopic sections (Kasthuri et al., 2015).

## Materials and Methods

### Obtaining Real Geometry of Tripartite Synapses

Real geometry of synapses and surrounding astrocytic processes were obtained from the high-resolution (6 × 6 × 30 nm) reconstruction of a 1,500 μm^3^ volume of mouse neocortex (Kasthuri et al., 2015), containing 1,700 identified and characterized synapses. In the first step, 208 “single” excitatory synapses with individual glutamatergic axon terminal synapsed to single postsynaptic dendritic spines were selected for simulation. Geometry of segmented cells in 1.2 × 1.2 × 1.2 μm volumes (201 × 201 × 41 pixels) around each post-synaptic density centroid were imported from the database to Matlab using the VAST Lite 1.2.1 software and custom-written Matlab scripts.

To correct geometry for fixation-induced swelling, we shrunk the segmented cells by 6 nm and extended the extracellular space (ECS) to this volume. This way, a fraction of the ECS in the synaptic environment was increased from 11.2 ± 3.0% to 18.2 ± 3.3% that is closer to physiological values (Van Harreveld and Khattab, 1968; Harreveld and Fifkova, 1975; Korogod et al., 2015; Pallotto et al., 2015).

Astrocytic coverage of the presynaptic axon terminal (bouton) and the postsynaptic dendritic spine was calculated by counting the number of surface pixels of boutons and spines having close contact with astrocytes. The surface/volume ratio (SVR) was determined by dividing the number of surface pixels counted according to the above method by the number of all pixels belonging to astrocyte processes.

### Simulation of Astrocytic [Ca^2+^] and Synaptic Glu Release

Extracellular concentrations of relevant ions ([Na^+^]_e_ = 140 mM; [K^+^]_e_ = 3 mM; [Ca^2+^]_e_ = 2 mM) as well as astrocytic [K^+^]_i_ (130 mM) and [Glu]_i_ (3 mM) were kept constant during the simulation, while [Glu]_e_ (0.3 μM), [Na^+^]_i_ (15 mM) and [Ca^2+^]_i_ (100 nM) were allowed to change due to Glu release, intracellular Ca^2+^ diffusion and activation of EAATs and NCX (Héja and Kardos, 2020). It is to note that [Glu]_e_ is difficult to measure and rather different estimates are reported in the literature. Electrophysiological measurements suggest tens of nanomolar concentrations (Herman et al., 2011) based on receptor activation, while microdialysis studies measure tens of micromolar for [Glu]_e_ (Baker et al., 2002). Furthermore, EC_50_ values of postsynaptic glutamate receptor (382 μM; Jonas and Sakmann, 1992; Li et al., 2002) and astrocytic glutamate transporter (14.8 μM; Levy et al., 1998; Herman and Jahr, 2007) indicate effective activation of postsynaptic receptors and extrasynaptic transporters at above 100 μM and 3 μM glutamate, respectively. These glutamate concentration ranges are far beyond the [Glu]_e_ of 0.4 ± 0.1 μM (Kékesi et al., 2000) allowing for receptor/transporter activation. Our *in vivo* microdialysis data also validates the mean of these values as being 0.4 ± 0.1 μM (Kékesi et al., 2000). Therefore, 0.3 μM [Glu]_e_, used in this study seems a reliable estimate.

Markovian kinetic models of astrocytic EAATs and NCX were constructed according to published rate constants based on experimental data. Glutamate uptake by EAATs was modeled by a 13-step cycle comprised of separate bindings and unbindings of 3 Na^+^, 1H^+^, 1 K^+^, and 1 Glu molecules (Bergles et al., 2002). NCX activity was modeled by a 6-step cycle according to Chu et al. (2016). 10,800/μm^2^ EAAT (Lehre and Danbolt, 1998) and 500/μm^2^ NCX (Chu et al., 2016) molecules were distributed randomly on the astrocytic surface.

Before starting the simulation, EAAT and NCX randomly populated the available states and we allowed them to reach steady-state distribution for 30 ms at the above concentrations. Simulations began with a further 10 ms baseline activity before initiating single synaptic glutamate release (5,000 Glu molecules) at the synapse centroid as determined by Kasthuri et al. (2015). The diffusion of independent glutamate molecules in the 3D ECS was estimated by random walks at 1 μs intervals. The diffusion coefficient of glutamate was set to 0.33 μm^2^/ms (Gavrilov et al., 2018).

**Scheme 1:**
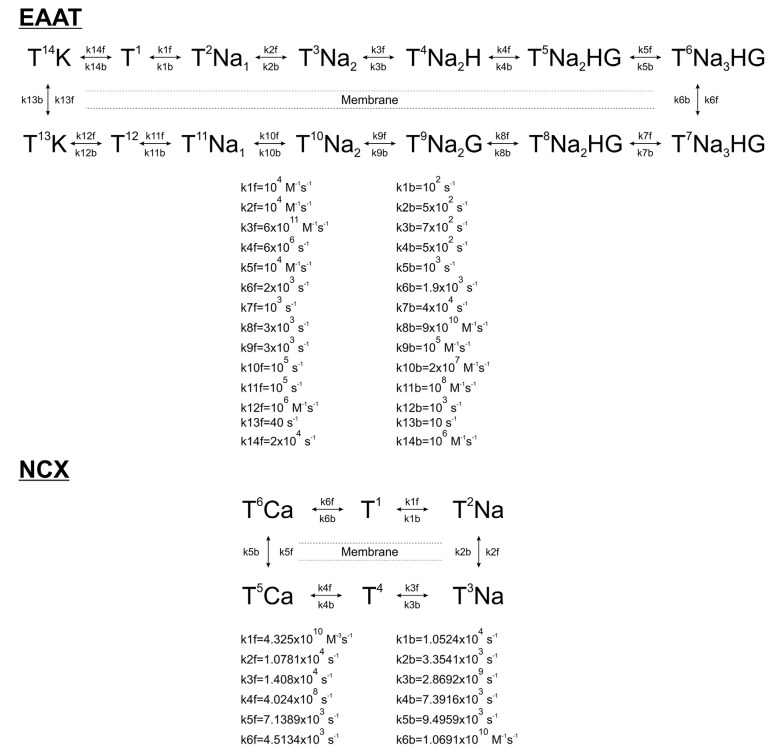
Kinetic schemes and rates of astrocytic Glu transporters (EAAT) and Na^+^/Ca^2+^ exchanger (NCX).

Each time steps (1 μs) was comprised of the following functions: (1) position of extracellular glutamate molecules and intracellular Ca^2+^ ions were updated by moving them with normally distributed random distances around their mean square displacement values. If a particle moved outside of the sample volume, it was removed from the available pool, except if [Glu]_e_, [Na^+^]_i_ or [Ca^2+^]_i_ dropped below the baseline level, in which case it was moved back to its previous position. Particles moving out of their compartment (astrocyte, dendrite, axon terminal, or ECS) were also placed back to their previous position. (2) Transition states of EAAT and NCX molecules were determined according to their rate constants and dynamic rate constants based on the current intra- and extracellular concentrations of relevant ions (Scheme 1). In the case of EAAT kinetics, local [Glu]_e_ in the surrounding 50 × 50 × 50 nm^3^ extracellular microdomain of each EAAT molecule was used instead of the average extracellular glutamate concentration. Local [Glu]_e_ was determined by counting the freely diffusing Glu molecules in the 50 × 50 × 50 nm^3^ ECS around each EAATs in each time frame. Transition rates were corrected for Q_10_ = 3 to account for temperature dependence. Astrocyte membrane potential was set to −70 mV. (3) Glutamate molecules bound to the extracellularly faced EAAT were removed from the available pool until they were released back by reverse operation of the transporter. Ca^2+^ ions bound to the intracellularly faced NCX were removed from the available pool until they were released back by reverse operation of the transporter.

All simulations were done in Matlab using custom-written scripts^1^. Reconstructed and segmented EM stacks of real synapses were downloaded and handled by the VAST Lite 1.2.1 software^2^ (Kasthuri et al., 2015) and the VastTools Matlab package. Processed data of synapses containing 3D geometries and calculated surfaces and volumes in Matlab file format as well as tools to reproduce the simulations can be downloaded at http://downloadables.ttk.hu/heja/Front_CellNeurosci2021. Synapses were visualized using Cinema4D.

Data are shown as mean ± SEM and were analyzed with one-way analysis of variances (ANOVAs, OriginPro 2018). Statistical significance was considered at *p* < 0.05.

## Results

To simulate Ca^2+^ oscillations in real astrocyte processes, we used the saturated reconstruction of a 1,500 μm^3^ volume of mouse neocortex (Kasthuri et al., 2015). The dataset contains 1,700 identified and morphologically characterized synapses. We explored volumes of 1.2 × 1.2 × 1.2 μm around these synapses to investigate the potential of astrocytic processes to readout synaptic activity.

Due to the applied glutaraldehyde and paraformaldehyde fixative, the ECS of the sample was found to occupy only 6% of the total volume around the synapses (Kasthuri et al., 2015). Since ECS fraction was found to be between 15% and 25% in frozen tissues (Van Harreveld and Khattab, 1968; Harreveld and Fifkova, 1975; Korogod et al., 2015; Pallotto et al., 2015) where fixation-issued swelling is not present, we modified the original segmentation by replacing the outer 6 nm surface of each cellular segment with ECS. This modification also allowed free diffusion of the released glutamate in the ECS, which would otherwise be hindered due to the direct connection of segmented cells.

To simulate spontaneous and glutamate-release associated, NCX activity-linked Ca^2+^ changes in real glutamatergic tripartite synapses, we selected 208 “classical” synapses out of the 1,700 identified synapses (Kasthuri et al., 2015) based on the following criteria: (1) axon type is excitatory; (2) axon terminal is present, i.e., it is not an en-passant synapse; (3) axon bouton is not multi-synaptic; (4) the postsynaptic element is a spine, not a shaft; and (5) astrocytic volume fraction is at least 2% in the 1.2 × 1.2 × 1.2 μm volume. Astrocytic Ca^2+^, extracellular glutamate concentrations following synaptic Glu release, as well as dynamics of astrocytic Glu transporters (EAAT) and NCX were simulated as previously described (Héja and Kardos, 2020).

**Figure 1:**
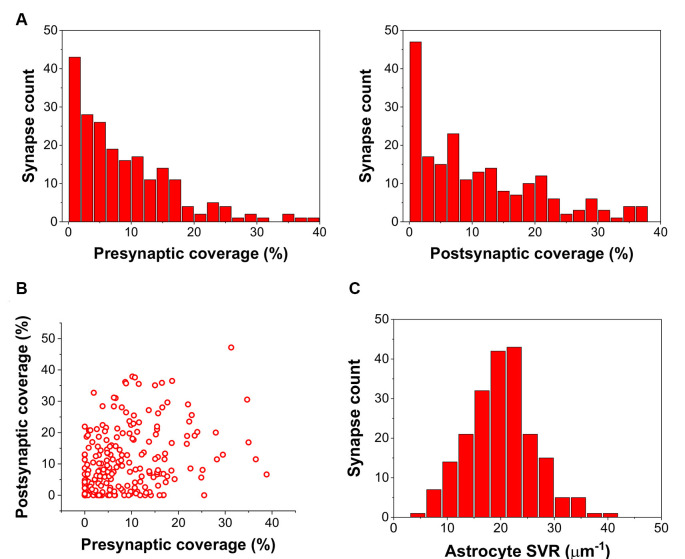
Astrocyte related geometric parameters of the 208 investigated synapses. **(A)** Distribution of pre-and postsynaptic coverage of synapses by astrocyte processes. **(B)** Correlation of pre-and postsynaptic coverage of synapses by astrocyte processes. **(C)** Distribution of the astrocytic surface/volume ratio (SVR) in the 1.2 × 1.2 × 1.2 μm volume surrounding each synapse.

By calculating the ratio of the axon terminal and spine surfaces that are in contact with astrocytic processes, we found many presynaptic axon terminals and postsynaptic spines with little or no astrocytic coverage at all (Figure 1A). Also, astrocytic coverage of pre- and postsynaptic elements showed a high degree of heterogeneity (Figure 1B). Although many of the synapses were equally covered by astrocytes at the axon terminal and the dendritic spine, highly asymmetric astrocytic coverage was also abundant. Besides, we also determined the surface to volume ratio (SVR) of astrocytic processes in the surrounding of the 208 selected synapses. Following previous observations (Gavrilov et al., 2018), the distribution of SVR followed normal distributions with a mean between 20 and 25 μm^−1^, corresponding to astrocytic leaflets that are known to cover synapses (Gavrilov et al., 2018; Figure 1C).

**Figure 2:**
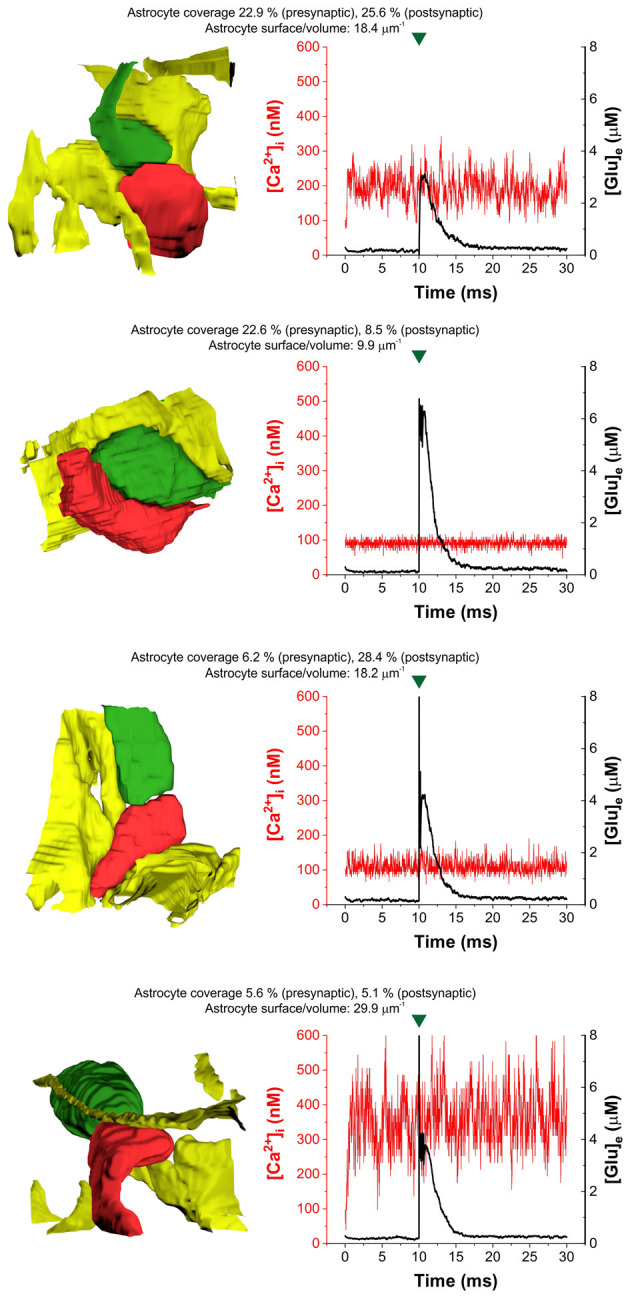
Heterogeneity of astrocytic intracellular Ca^2+^ oscillations ([Ca^2+^]i) during the control period (1–10 ms) and following glutamate release (10–30 ms) in four selected real tripartite synapses showing different levels of astrocytic coverage and astrocyte SVR. The dynamics of astrocytic Ca^2+^ concentration is shown in red, extracellular [Glu] ([Glu]_e_) is shown in the black trace. Astrocytes are colored yellow, presynaptic terminals are green and postsynaptic spines are red in images showing real 3D geometry of each synapse. Green marks indicate release events.

In agreement with previous findings (Héja and Kardos, 2020), we found that astrocytic oscillatory Ca^2+^ dynamics spontaneously emerged in different kinds of realistic astrocytic leaflets characterized by various pre- or postsynaptic contacts (Figure 2). The incidence of Ca^2+^ fluctuations strongly depends on the astrocytic SVR and also correlates with pre- and postsynaptic astrocytic coverage (Figure 2). High astrocytic SVR frequently correlated with large amplitude fluctuations of astrocytic Ca^2+^ concentration both spontaneously and following glutamate release (Figure 2). Medium SVR in conjunction with high coverage of both presynaptic axon terminal and postsynaptic dendritic spine is characterized by the medium intensity of Ca^2+^ fluctuations that is unaffected by glutamate release (Figure 2). On the other hand, no astrocytic Ca^2+^ fluctuations emerge at low SVR (Figure 2).

**Figure 3:**
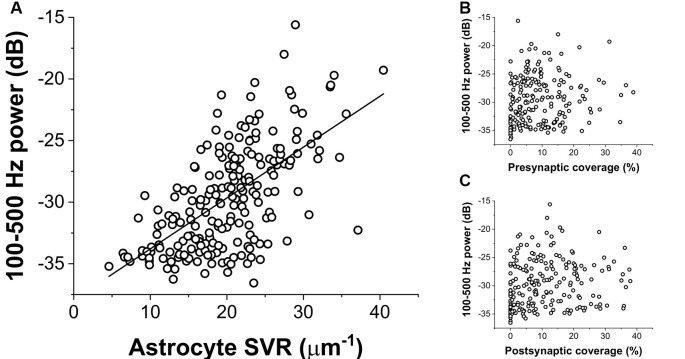
Correlation of the power of astrocytic Ca^2+^ oscillations (100–500 Hz) with geometric characteristics of the astrocyte processes. **(A)** Ca^2+^ oscillation is more pronounced in thin astrocytic processes characterized by a high SVR. The black line shows linear regression fitted to the data (*R*^2^ = 0.38). **(B,C)** Astrocytic Ca^2+^ oscillation shows no correlation with the extent of pre-or postsynaptic coverage by astrocytes.

To quantify the extent of NCX-mediated astrocytic Ca^2+^ oscillations, we calculated the power spectral density of the Ca^2+^ signal and summed its power in a wide range between 100 and 500 Hz. The power of these high-frequency Ca^2+^ oscillations showed a direct correlation with increasing SVR, i.e., it is more apparent in thin astrocytic processes (Figure 3A). By contrast, the power of high-frequency Ca^2+^ fluctuations does not depend on either pre- or postsynaptic astrocytic coverage (Figures 3B,C).

**Figure 4:**
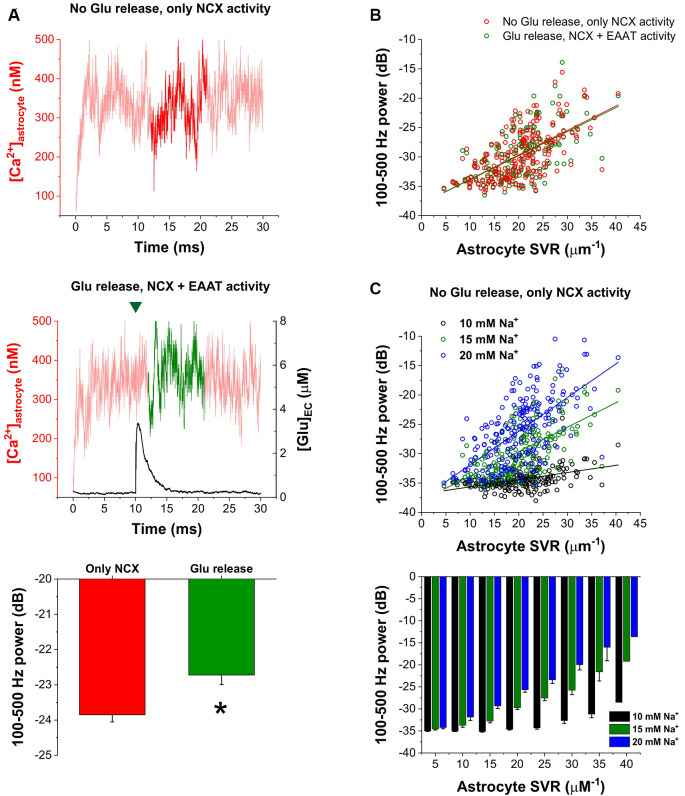
Increased astrocytic Na^+^ concentration enhances astrocytic Ca^2+^ oscillations. **(A)** Representative traces of astrocytic Ca^2+^ concentration in the astrocytic processes surrounding a synapse with high SVR (22.4 μm^−1^). Dark traces represent the analyzed period during which 100–500 Hz power was calculated when no glutamate release occurred and only NCX activity was allowed (*top*) or when 5,000 molecules of glutamate were released at 10 ms. The green mark indicates the release event (*middle*). Power of the 100–500 Hz range increased from −23.85 ± 0.20 dB to −22.72 ± 0.27 dB due to synaptic glutamate release in this synapse (*n* = 5 simulation runs, *p* = 0.01; asterisk means significant difference, *bottom*). **(B)** Correlation of 100–500 Hz powers with astrocytic SVR with (green) and without (red) synaptic glutamate release in the 12–21 ms period in all the investigated 208 synapses. Green and red lines show linear regression fitted to the data (*R*^2^ = 0.38 for both lines). **(C)** Correlation of 100–500 Hz powers with astrocytic SVR at different astrocytic Na^+^ concentrations in the 12–21 ms period in all the investigated 208 synapses. No glutamate release was initiated, only NCX activity was considered. Black, green and blue lines show linear regression fitted to the data (*R*^2^ = 0.20, *R*^2^ = 0.38 and *R*^2^ = 0.39 respectively; *top*). Grouping of power vs. astrocyte SVR data shows that increasing astrocytic Na^+^ concentration enables the emergence of Ca^2+^ oscillations in thicker processes (*bottom*).

Furthermore, we also investigated whether synaptic glutamate release alters the spontaneous NCX-mediated Ca^2+^ fluctuations. To this end, we compared the oscillatory powers of the Ca^2+^ concentration signals in the 100–500 Hz range in two different conditions: (1) simulating baseline Ca^2+^ fluctuations when only NCX was allowed to operate and no synaptic glutamate release occurred; and (2) simulating Ca^2+^ fluctuations according to our original conditions, releasing 5,000 glutamate molecule after 10 ms of baseline activity and letting EAATs function. The powers of the 100–500 Hz range of the Ca^2+^ concentration signals were compared in the 12–21 ms period. In some synapses, glutamate release significantly increased the 100–500 Hz power. As an example, 100–500 Hz power increased from −23.85 ± 0.20 dB to −22.72 ± 0.27 dB due to synaptic glutamate release (*n* = 5 simulation runs, *p* = 0.01) in a synapse with high SVR (22.4 μm^−1^; Figure 4A). However, although a slight increase was also observed on the population level, this increase was not significant (−29.73 ± 0.29 dB vs. −29.61 ± 0.29 dB, *n* = 208 synapses, *p* = 0.09). Therefore, we investigated whether synapses characterized by different SVR of the surrounding astrocytes may respond differently to Glu release. Resolution of Glu release-induced changes in the power of astrocytic Ca^2+^ fluctuations by astrocyte SVRs, however, still did not reveal a significant effect of Glu release (Figure 4B). Since single Glu release events only slightly increase astrocytic Na^+^ concentration, we investigated whether more pronounced (but still physiological) changes in astrocytic Na^+^ concentration may significantly affect NCX-mediated Ca^2+^ fluctuations. Changing astrocytic Na^+^ concentration from the original 15 mM to 10 or 20 mM, indeed, markedly altered Ca^2+^ oscillatory power (Figure 4C). Increasing astrocytic Na^+^ concentration enhances Ca^2+^ fluctuations in general, and consequently allows the emergence of such oscillations in thicker processes characterized by smaller SVR.

## Discussion

Spontaneous astroglial Ca^2+^ fluctuations, mediated by NCX in real excitatory tripartite synapses appear to be primarily dependent on astrocytic SVR. In our simulations, more pronounced NCX-operated Ca^2+^ fluctuations are associated with high SVR, suggesting that thin astrocytic processes are capable to spontaneously generate astrocytic Ca^2+^ signals. Although, we found that NCX mediated spontaneous Ca^2+^ fluctuations are not significantly modulated by single Glu release events and corresponding Na^+^ entry through plasma membrane glutamate transporters, we showed that increasing astrocytic Na^+^ concentration in the physiological range markedly enhances Ca^2+^ fluctuations in real tripartite synapses, especially in those, characterized by high SVR. Therefore, we hypothesize that bursting synaptic activity or simultaneous activation of multiple synapses in the domain of a single astrocyte may significantly contribute to the emergence and enhancement of Ca^2+^ fluctuations by increasing astrocytic Na^+^ concentration. The scenario with Na^+^ threshold and mechanistic explanation, however, remains to be clarified. Importantly, astrocytic Ca^2+^ concentration can also be directly increased by the activation of astrocytic NMDA receptors (Ziemens et al., 2019) that are currently not included in our model.

It is evident from our simulations that the appearance of fast Ca^2+^ fluctuations is correlated to the high surface-to-volume ratio of PAPs. Unfortunately, neither spatial nor temporal resolution of current experimental techniques allows the direct observation of such fast (>100 Hz) Ca^2+^ signals in tiny processes (*d* < 2–300 nm, SVR > 10; Rusakov, 2015). Therefore, we can only speculate about how these spontaneous Ca^2+^ events, triggered by Ca^2+^ entry through NCX can propagate into astrocytic branchlets and can be amplified and propagated as a result of various downstream mechanisms, including Ca^2+^-dependent Ca^2+^ release in association with activation of inositol 1,4,5-trisphosphate receptors (IP_3_R) or mitochondrial permeability transition pores (Semyanov et al., 2020). It was experimentally observed, however, that the appearance and frequency of slower spontaneous Ca^2+^ events in somewhat larger astrocytic processes (characterized by SVR < 3) depend on SVR (Wu et al., 2019). Also, compartmentalized Ca^2+^ waves as predicted by the dynamically rich repertoire of distinct Ca^2+^-dependent Ca^2+^ release dynamics (Matrosov et al., 2019) may travel and act by modulating local spontaneous Ca^2+^ fluctuations. Indeed, the shape of the slow Ca^2+^ wave with fast Ca^2+^ fluctuations (Savtchenko et al., 2018; SI Figure 12) may indicate the superimposition of slow waves and fast Ca^2+^ fluctuations locally. It is to mention, that fast astrocytic Ca^2+^ signaling with mean onset time as rapid as that of neurons is not unprecedented (Kékesi et al., 2015; Pál et al., 2015; Lind et al., 2018; Stobart et al., 2018; Semyanov et al., 2020). Assessing the true impact of spontaneously emerging, local high-frequency Ca^2+^ fluctuations on the evolution of cellular- and network-scale Ca^2+^ oscillations necessitates further studies, that include models describing downstream Ca^2+^ stores and Ca^2+^ buffers (Savtchenko et al., 2018; Matrosov et al., 2019), as well as simulate Ca^2+^ dynamics in multiple, neighboring synapses contacted by the same astrocyte. We may also conjecture that the structural plasticity of astrocytic processes may serve as a *de novo* signal generator, independently of its role in regulating glutamate spillover, K^+^ buffering, or other indirect forms of modulation of neuronal activity (Henneberger et al., 2020). These findings suggest a prominent role for dynamically changing PAPs in neuro-glial coupling.

## Data Availability Statement

Raw data is available to download at http://downloadables.ttk.hu/heja/Front_Cell_Neurosci_2021. Matlab scripts used to process the data can be downloaded at https://github.com/hejalaszlo/Astrocyte-leaflet-simulation.

## Author Contributions

LH: conceptualization, data curation, formal analysis, funding acquisition, methodology, software, supervision, validation, visualization, roles/writing—original draft, writing—review and editing. ZS and MP: data curation, methodology, validation, roles/writing—original draft, writing—review and editing. JK: conceptualization, investigation, methodology, supervision, roles/writing—original draft, writing—review and editing. All authors contributed to the article and approved the submitted version.

## Conflict of Interest

The authors declare that the research was conducted in the absence of any commercial or financial relationships that could be construed as a potential conflict of interest.

## Footnotes

^1^https://github.com/hejalaszlo/Astrocyte-leaflet-simulation

^2^https://lichtman.rc.fas.harvard.edu/vast/
